# Potential Detrimental Interactions Between Metformin and Supplemental Dietary Fiber in Type 2 Diabetes

**DOI:** 10.1111/1753-0407.70101

**Published:** 2025-05-21

**Authors:** Rosemary M. Hall, Amber Parry‐Strong, David O'Sullivan, Jeremy D. Krebs, Olivier Gasser

**Affiliations:** ^1^ Department of Medicine University of Otago Wellington Wellington New Zealand; ^2^ Centre of Endocrine, Diabetes and Obesity Research (CEDOR) Wellington Wellington New Zealand; ^3^ Malaghan Institute Wellington New Zealand; ^4^ High‐Value Nutrition National Science Challenge Auckland New Zealand

**Keywords:** fiber, HbA1c, interactions, metformin, T2DM

**Summary**

- Despite clear evidence that dietary fiber reduces development of diabetes and associated complications, many consume inadequate amounts.
- Additional dietary fiber, provided to people with pre‐diabetes and T2DM and habitual low fiber intake, improved BMI and LDL cholesterol.
- However, a variable HbA1c response was observed: a reduction in those not taking Metformin and an increase in the small group using metformin alone.
- The interaction between metformin and supplemental fiber needs to be clarified and, in the meantime, used together with caution.

Higher intakes of dietary fiber have been associated with a reduced risk of developing Type 2 Diabetes (T2DM) and cardiovascular disease [1, 2]. Fiber supplementation improves overall glycaemia, with reductions in Hba1c and better insulin sensitivity [3]. However, there is significant reported heterogeneity on the effects of supplemental fiber on glycaemic outcomes for people with T2DM, potentially due to variations in absorption and metabolism. These differences, we believe, are worth examining more closely, especially considering the complexities of the gut microbiome, medications used in T2DM, and the role of the background diet [3].

In our recent study, we investigated the impact of supplemental fiber on glucose metabolism and glycemic control in people with pre‐diabetes and T2DM who had a low habitual fiber intake. We recruited 30 participants with HbA1c levels ranging from 45 to 70 mmol/mol, and provided them with psyllium fiber supplements for 12 weeks.

Although we observed reductions in body mass index (BMI) and improvements in lipid profiles, HbA1c levels did not significantly improve overall. Surprisingly, participants taking metformin alone experienced an increase in HbA1c, while those not taking metformin experienced a slight reduction (Figure 1).

**FIGURE 1:**
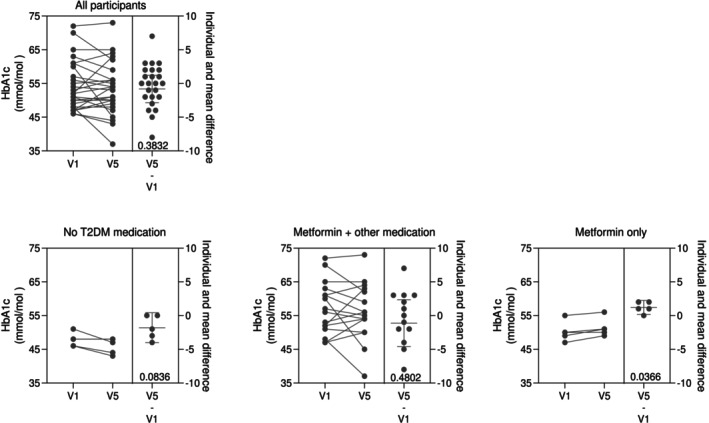
Changes in HbA1c after the 12 week fiber supplement stratified by medication type. Overall HbA1c reduced from 54 (7.33) to 52.2 (7.73) mmol/mol. In those taking metformin alone (*n* = 5) HbA1c increased from 50.2 to 51.4 mmol/mol. In those not taking T2DM medication (*n* = 5) HbA1c fell from 47.8 to 46.0 mmol/mol.

This discrepancy points to a critical issue: the potential interaction between metformin and fiber supplementation. Metformin, the most common medication for T2DM works by reducing hepatic glucose production and improving insulin sensitivity [4]. However, metformin primarily acts within the gastrointestinal tract, commonly producing gastrointestinal side‐effects and may alter gut microbiome, with the potential to directly affect fiber absorption [5]. Our findings suggest that when combined with fiber supplementation, metformin may impair the metabolic benefits typically associated with fiber, alongside a potential detrimental effect on the glycaemic benefits of metformin.

This phenomenon is consistent with previous research. For example, a study by Tramontana et al. found that a high‐fiber diet did not improve HbA1c in 78 patients with T2DM on metformin monotherapy [6], while other studies observed more promising results when fiber was combined with different medications [7]. These findings highlight the need for a more nuanced understanding of how dietary fiber interacts with both the gut microbiome and medications like metformin.

Moreover, the gut microbiota's role in metabolism is integral to the overall metabolic benefits. Dietary fiber, especially in the form of psyllium, is fermented by gut bacteria into short‐chain fatty acids (SCFAs), which have been shown to improve immune function and reduce inflammation—factors that are crucial in managing T2DM [7, 8]. However, both metformin and fiber alter the gut microbiome in different ways, and this complex interaction may undermine the potential benefits of both fiber supplementation and metformin for some individuals [8, 9].

Given these unexpected findings, which may have clinically important implications, we urge caution when recommending fiber supplementation for people with T2DM on metformin. While a high habitual intake of dietary fiber may provide metabolic benefits, it is essential to better understand how supplemental fiber interacts with metformin. Until this interaction is clarified, healthcare providers should carefully consider the use of fiber supplements in this population.

## Author Contributions

The protocol for this study was developed by all the authors. Amber Parry‐Strong (A.P.S.) recruited participants and performed study visits. Rosemary Hall (R.H.) and Jeremy Krebs (J.K.) provided clinical expertise for diabetes care. David O'Sullivan (D.O'S.) performed laboratory analysis of the samples and statistical analysis of the data. All authors contributed to data interpretation. RH wrote the manuscript and all authors contributed to the manuscript review.

## Conflicts of Interest

The authors declare no conflicts of interest.
